# Impact of levosimendan on efficacy and renal function in acute heart failure according to renal function: A perspective, multi-center, real-world registry

**DOI:** 10.3389/fcvm.2022.986039

**Published:** 2022-10-19

**Authors:** Han Zhang, Li Jiang, Rui Fu, Ping Qin, Xuan Zhang, Tao Tian, Guang-xun Feng, Yan-min Yang

**Affiliations:** Emergency Center, Fuwai Hospital, State Key Laboratory of Cardiovascular Disease, National Center for Cardiovascular Diseases, Chinese Academy of Medical Sciences and Peking Union Medical College, Beijing, China

**Keywords:** acute heart failure, levosimendan, efficacy, renal function, estimate glomerular filtration rate

## Abstract

**Objective:**

Acute heart failure (AHF) is associated with high mortality. Levosimendan, an inodilator, has proved to increase cardiac output and exert renoprotective effect in AHF. Our aim was to investigate the efficacy and renoprotective effects of levosimendan in patients with AHF and different renal function.

**Methods:**

This is a prospective, observational, multi-center registry. Patients admitted with AHF between June 2020 and May 2022 and treated with levosimendan during the hospital stay were included. Baseline characteristics, laboratory tests, electrocardiogram (ECG), chest X-ray, echocardiography, and treatment were collected. A 5-point Likert scale was used to document patients' baseline dyspnea. The estimated glomerular filtration rate (eGFR) was calculated by means of the Modification of Diet in Renal Disease equation. After levosimendan infusion, patients underwent assessment of degree of dyspnea, and levels of brain-type natriuretic peptide (BNP) /N-terminal pro-BNP (NT-pro BNP), and eGFR repeatedly.

**Results:**

Among 789 AHF patients who received levosimendan treatment in this study, 33.0 % were female, mean age was 64.9 ± 16.8 years, and mean eGFR was 72.6 ± 32.5 ml/min/m^2^. The mean score of dyspnea was 3.0 ± 1.0 using 5-point Likert scale before levosimendan infusion. Dyspnea improved in 68.7% patients at 6h after infusion of levosimendan, and in 79.5% at 24 h. Lower eGFR was associated with lower efficacy rate after 6h infusion (71.7, 70.7, 65.2, and 66.0%, respectively) and after 24 h infusion (80.5, 81.4, 76.2, and 77.8%, respectively). The levels of BNP or NT-pro BNP were also decreased after levosimendan treatment, and in each eGFR category. Levels of eGFR increased from baseline (72.6 ± 32.5 ml/min/m^2^) to 12–24h (73.8 ± 33.5 ml/min/m^2^) and 24–72h (75.0 ± 33.4 ml/min/m^2^) after starting treatment (*p* < 0.001). However, the eGFR levels increased only in patients with eGFR lower than 90.0 ml/min/m^2^.

**Conclusions:**

In AHF patients who received levosimendan, degree of dyspnea and levels of BNP or NT-pro BNP were significantly improved, especially in patients with higher eGFR levels. However, levosimendan infusion increase eGFR only in AHF patients with renal dysfunction.

## Introduction

Acute heart failure (AHF) is a major public health problem worldwide, defined as a status of acute-onset or rapidly worsening heart failure symptoms. AHF is a leading cause of hospitalizations in subjects aged >65 years and associated with high mortality and rehospitalization rates (1). Renal impairment is frequently observed in patients with AHF. When present, both entities relate to strongly impaired survival (2, 3). For AHF patients with low cardiac output and hypoperfusion, inotropes, such as norepinephrine, dopamine, and dobutamine are frequently used to improve cardiac output. Traditional inotropes for AHF is favorable to acute hemodynamic effects, but have no evidence of benefit on outcomes or renal function (4).

Levosimendan, first introduced as an inotropic agent for short-term treatment of AHF, also shows vasodilatory, and organ-protective properties introduced by open adenosine triphosphate-sensitive potassium (KATP) channels (5). Since the LIDO trial in 2002 (6), several studies have reported that levosimendan exert renal-protective effects *via* increase cardiac output and dilate predominantly the preglomerular afferent arterioles (7–10). However, clinical evidence from randomized controlled trials (RCTs) is divergent due to small sample sizes and heterogeneity across studies. Furthermore, the renoprotective effect of levosimendan in real-word AHF patients with different renal function is limited.

In this study, we investigated the renoprotective effects of levosimendan in AHF patients with different renal function, as well as the efficacy on dyspnea and levels of brain-type natriuretic peptide (BNP) or elevated N-terminal pro-BNP (NT-pro BNP) in a real-word registry.

## Methods

### Study participants

In this prospective, multicenter, observational study, patients admitted for AHF at 20 hospitals in Beijing, between June 2020 and May 2022 and were treated with levosimendan during the hospital stay. In accordance with the guidelines (1), AHF was diagnosed on the signs and symptoms, symptomatic lung congestion confirmed by chest X-ray, or structural and functional abnormalities on echocardiography, elevated brain-type natriuretic peptide (BNP) or elevated N-terminal pro-BNP (NT-pro BNP). There were no specific exclusion criteria. The date of entry in the study was defined as the day of the first levosimendan infusion during the hospital stay.

### Ethical approval

The Institutional Review board of Fuwai Hospital (China) approved this study. The study design follows the guidelines of the Declaration of Helsinki. Data were collected only after detailed information regarding the study was provided and written informed consent was obtained from all participants.

### Data collection and procedures

Data were collected with the use of case report forms (CRFs) and entered into a web-based system. Baseline characteristics, including age, sex, past medical history, New York Heart association (NYHA) functional classification, electrocardiogram (ECG), chest X-ray, and comorbidities. Laboratory tests (including blood routine, liver and renal function, electrolytes, troponin, creatine kinase - MB, and natriuretic peptide levels) and echocardiography were performed at admission. estimated glomerular filtration rate (eGFR) was calculated by the Chinese-modified Modification of Diet in Renal Disease (MDRD) equation using four variables (11).

Treatment before and during hospitalization, including pharmacological and non-pharmacological management, were collected. A 5-point Likert scale was used to document patients' baseline status: (i) not short of breath, (ii) mildly short of breath, (iii) moderately short of breath, (iv) severely short of breath, and (v) very severely short of breath. Study drug, levosimendan (12.5 mg/5ml, Qilu Pharma, Shandong, China) was administrated at the discretion of clinicians, based on clinical assessment of patients. The loading and maintenance dose of levosimendan was titrated according to the hemodynamic effect (5).

At 6 h and 24 h after levosimendan infusion, patients underwent assessment of degree of dyspnea to define the efficacy of levosimendan infusion. A 7-point Likert scale administered at 6 h or 24 h after infusion was used to determine change from baseline: (i) markedly worse, (ii) moderately worse, (iii) slightly worse, (iv) no change, (v) slightly improved, (vi) moderately improved, and (vii) markedly improved. The scale “markedly worse”, “moderately worse”, and “slightly worse” was defined as exacerbated. The term “efficacy” was defined as the percentage of patients assessed as “markedly improved”, “moderately improved” and “slightly improved” in the total number of patients subjected to the efficacy analysis. Furthermore, parameters of renal function and BNP or NT-pro BNP levels were repeatedly measured at 12–24 h and 24–72 h after the administration of levosimendan, to examine the impact of levosimendan on renal function.

Adverse events during levosimendan infusion were recorded, including: hypotension (defined as systolic blood pressure <80 mmHg and/or lowering by more than 40 mmHg compared with the start of levosimendan therapy), new onset arrhythmia needs medical treatment and other potential adverse events (headache, nausea, vomiting and pruritus, etc.).

### Statistical analysis

Continuous normally distributed data were summarized using mean ± SD (standard error), and non-normally distributed continuous data are presented as median (interquartile range [IQR]). Normal distribution of continuous data was checked using histograms. Categoric variables were presented as number and percentages. Baseline characteristics were assessed using the chi-squared test for dichotomous variables and independent samples *t*-test or Mann-Whitney *U* test for continuous variables. Changes in renal function and BNP/NT-pro BNP before and after levosimendan administration were assessed by repeated-measures ANOVA. Statistical significance was defined as *P* < 0.05 (2-tailed). All statistical analyses were performed using SPSS 25.0 (SPSS Inc., Chicago, IL, USA).

## Results

### Baseline characteristics

Between June 2020 and May 2022, a total of 800 AHF patients received levosimendan infusion during the hospitalization in 20 centers, Beijing. Of these, 11 patients were excluded because of missing data, the remaining 789 patients were included to analyze.

Baseline characteristics according to the eGFR level are presented in Table 1. Of the 789 patients, 33.0% were female, mean age was 64.9 ± 16.8 years, and 75.0% had a previous history of heart failure. The most common etiology of AHF was ischemic heart disease in 50.6%, followed by cardiomyopathy (33.3%) and valvular heart disease (8.0%). To assess the severity of acute heart failure, we used the Killip classification in AHF patients caused by acute myocardial infarction (AMI) (*N* = 166), and the NYHA (New York Heart Association) classification in those without AMI (*N* = 623). The mean LVEF (Left ventricular ejection fraction) was 35 ± 11%. Common comorbidities included hypertension (54.8%), coronary heart disease (46.3%), and diabetes mellitus (36.0%). During hospital stay, loop diuretics, angiotensin-converting enzyme inhibitors (ACEI)/angiotensin II receptor blockers (ARB)/angiotensin receptor- neprilysin inhibitor (ARNI), β-blockers, mineralocorticoid receptor, vasodilators and inotropes were used in 85.0, 58.2, 73.0, 50.3, 61.9, and 55.5%, respectively.

**Table 1:** Baseline characteristics of 789 AHF patients according to eGFR level.

**Characteristic**	**Total** **(*N =* 789)**	**GFR, ≥90.0 ml/min/1.73 m^2^ (*N =* 210)**	**eGFR, 60.0–89.9 ml/min/1.73 m^2^ (*N =* 266)**	**eGFR, 30.0–59.9 ml/min/1.73 m^2^ (*N =* 259)**	**GFR, < 30.0 ml/min/1.73 m^2^ (*N =* 54)**	***p–*Value**
Age, yr	64.9 ± 16.8	61.4 ± 17.6	63.9 ± 17.3	67.2 ± 15.4	73.2 ± 12.9	< 0.001
Female sex, *n* (%)	260 (33.0)	56 (26.7)	75 (28.2)	100 (38.6)	29 (53.7)	< 0.001
Body mass index, kg/m^2^	24.1 ± 4.3	24.6 ± 4.8	24.2 ± 3.9	23.6 ± 4.3	24.3 ± 4.4	0.084
Blood pressure, mm Hg						
Systolic	119.0 ± 23.1	119.9 ± 23.8	119.2 ± 21.7	117.4 ± 23.5	121.6 ± 25.6	0.516
Diastolic	72.9 ± 14.8	72.2 ± 13.5	74.5 ± 15.5	72.2 ± 14.6	71.3 ± 16.2	0.204
Heart rate, bpm	87.7 ± 21.2	88.4 ± 21.7	88.2 ± 21.1	86.7 ± 21.3	88.4 ± 19.9	0.810
Previous heart failure, *n* (%)	592 (75.0)	135 (64.6)	206 (77.5)	209 (80.8)	42 (78.3)	0.001
**Etiology of AHF**, ***n*** **(%)**						0.036
Ischemic heart disease	399 (50.6)	116 (55.2)	126 (47.4)	122 (47.1)	35 (64.8)	
Cardiomyopathy	263 (33.3)	61 (29.0)	99 (37.2)	88 (34.0)	15 (27.8)	
Valvular heart disease	63 (8.0)	17 (8.1)	14 (5.3)	30 (11.6)	2 (3.7)	
Other cause	64 (8.1)	16 (7.6)	27 (10.2)	19 (7.3)	2 (3.7)	
**Comorbidities**, ***n*** **(%)**						
Coronary artery disease	365 (46.3)	86 (41.0)	108 (40.6)	141 (54.4)	30 (55.6)	0.002
Old myocardial infarction	179 (22.7)	42 (20.0)	54 (20.3)	72 (27.8)	11 (20.4)	0.124
Hypertension	432 (54.8)	111 (52.9)	132 (49.6)	151 (58.3)	38 (70.4)	0.021
Diabetes mellitus	284 (36.0)	68 (32.4)	87 (32.7)	110 (42.5)	19 (35.2)	0.067
Dilated cardiomyopathy	185 (23.4)	43 (20.5)	70 (26.3)	59 (22.8)	13 (24.1)	0.501
Valvular disease	87 (11.0)	29 (13.8)	20 (7.5)	35 (13.5)	3 (5.6)	0.056
Chronic renal dysfunction	174 (28.8)	11 (6.1)	30 (15.5)	102 (55.4)	31 (72.1)	< 0.001
Stroke	103 (13.1)	16 (7.6)	28 (10.5)	42 (16.2)	17 (31.5)	< 0.001
Current smoker	103 (13.1)	39 (18.6)	37 (13.9)	25 (9.7)	2 (3.7)	0.011
Current alcoholic	81 (10.3)	33 (15.7)	27 (10.2)	20 (7.7)	1 (1.9)	0.011
**Baseline cardiac function**						
NYHA class (III or IV) ^*^	547 (87.8)	126 (82.9)	186 (87.3)	200 (91.0)	35 (92.1)	0.025
Killip class (III or IV)^†^	66 (39.7)	24 (41.4)	18 (33.9)	14 (35.9)	10 (62.5)	0.438
Degree of dyspnea^‡^	3.0 ± 1.0	3.0 ± 1.0	2.9 ± 1.0	3.3 ± 0.9	3.1 ± 0.9	< 0.001
Lung congestion on X–ray	670 (84.9)	173 (82.4)	228 (85.7)	219 (84.6)	50 (92.6)	0.296
**Electrocardiograph at admission**						
QRS width, ms	112 ± 26	109 ± 25	111 ± 24	115 ± 27	111 ± 30	0.463
QTc width, ms	434 ± 54	420 ± 55	439 ± 48	440 ± 54	444 ± 60	0.004
Atrial fibrillation/flutter	252 (31.9)	45 (21.4)	88 (33.1)	95 (36.7)	24 (44.4)	0.001
**Echocardiography**						
Left atrial diameter, mm	46 ± 10	44 ± 9	46 ± 10	47 ± 10	46 ± 11	0.120
LV end-diastolic diameter, mm	60 ± 12	59 ± 12	61 ± 13	60 ± 12	57 ± 12	0.117
LVEF, %	35 ± 11	36 ± 11	35 ± 11	35 ± 11	37 ± 12	0.353
**Baseline laboratory data**						
Hemoglobin, g/dl	12.2 ± 2.6	12.7 ± 2.4	12.5 ± 2.6	11.6 ± 2.5	10.8 ± 2.6	0.001
Alanine aminotransferase, U/L	48.1 ± 102.1	43.1 ± 64.0	59.1 ± 133.0	41.9 ± 95.7	44.7 ± 79.9	0.230
BNP, pg/mL^$^	789 (483–1,722)	488 (283–931)	789 (521–1,558)	1,355 (560–2,560)	1,532 (947–2,760)	0.003
NT-pro BNP, pg/mL^#^	6,235 (3,200–13,314)	4,260 (1,935–7,764)	5,639 (3,121–10,477)	8,792 (4,414–18,725)	13,314 (6,335–25,827)	< 0.001
Sodium, mmol/L	138.86 ± 5.29	139.14 ± 5.48	138.73 ± 4.84	138.53 ± 5.05	139.59 ± 7.09	0.612
Potassium, mmol/L	4.11 ± 0.55	4.04 ± 0.51	4.09 ± 0.52	4.16 ± 0.57	4.27 ± 0.65	0.014
Serum creatinine, μmol/L	112.0 ± 64.9	77.0 ± 58.5	98.9 ± 26.8	130.4 ± 44.2	222.8 ± 125.0	< 0.001
eGFR, ml/min/1.73m^2^	72.3 ± 32.1	114.1 ± 21.0	74.8 ± 8.8	46.0 ± 8.8	23.1 ± 6.0	< 0.001
**Medications**, ***n*** **(%)**						
Loop diuretics	671 (85.0)	167 (79.5)	225 (84.5)	236 (91.1)	43 (79.6)	0.004
ACEI/ARB/ARNI	459 (58.2)	115 (54.8)	167 (62.8)	156 (60.2)	21 (38.9)	0.019
β-blockers	576 (73.0)	142 (67.6)	201 (75.6)	195 (75.3)	38 (70.4)	0.219
MRA	397 (50.3)	80 (38.1)	147 (55.3)	151 (58.3)	19 (35.2)	< 0.001
Vasodilators	488 (61.9)	112(53.3)	167 (62.8)	177 (68.3)	32 (59.3)	0.019
Inotropes	438 (55.5)	99 (47.1)	149 (56.0)	156 (60.2)	34 (63.0)	0.027

Data are reported as median (interquartile range) for the continuous variables, or as *n* (%) for the categorical variables.

* Data were available in 166 patients with acute myocardial infarction in the overall cohort;

† Data were available in 623 patients without acute myocardial infarction in the overall cohort;

‡ The 5-point of the Likert scale received a number from 0 (no dyspnea) to 4.

$ Data were available in 83 patients in the overall cohort;

# Data were available in 691 patients in the overall cohort.

AHF, Acute heart failure; eGFR, estimated glomerular filtration rate; NYHA, New York Heart Association; LVEF, Left ventricular ejection fraction; BNP, Brain natriuretic peptide; NT-pro BNP, N-terminal pro-brain natriuretic peptide; BUN, Blood urea nitrogen; ACEI, denotes angiotensin-converting enzyme inhibitor; ARB, angiotensin II receptor blocker; ARNI, angiotensin receptor- neprilysin inhibitor; MRA, mineralocorticoid receptor antagonist.

**Figure 1:**
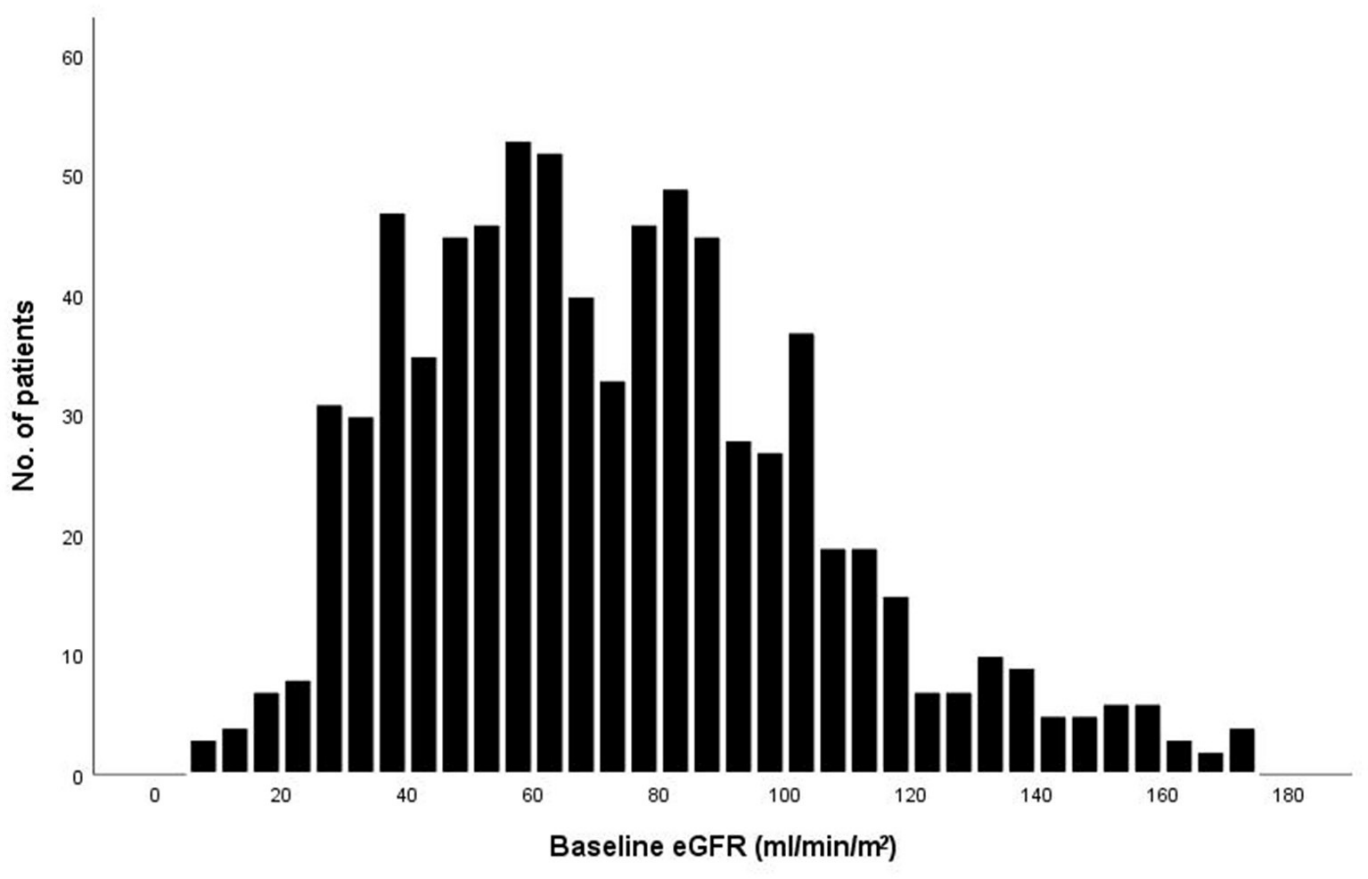
Distribution of eGFR at baseline among the 789 patients.

The baseline eGFR of the 789 patients was normally distributed (Figure 1). The mean (±SD) eGFR was 72.3 ± 32.1 ml/min/m^2^ (range from 8.5 to 174.1), 210 (26.6%) had an eGFR more than 90 ml/min/m^2^, 266 (33.7%) had an eGFR of 60.0 to 89.9 ml/min/m^2^, 259 (32.8%) had an eGFR of 30.0 to 59.9 ml/min/m^2^, and 54 (6.8%) had an eGFR of less than 30.0 ml/min/m^2^. Patients with a lower eGFR was associated with increasing age and female sex, although these variables were used in the determination of eGFR. The proportions of patients with NYHA function class III – IV and degree of dyspnea at baseline, as well as levels of Brain natriuretic peptide (BNP) or N-terminal pro- brain natriuretic peptide (NT-pro BNP), increased with decreasing eGFR (Table 1). Patients in the lowest category of eGFR had the highest rates of hypertension, prior coronary artery disease, diabetes mellitus, and prior stroke. The proportions of patients who were receiving initial pharmacotherapies (diuretics, ACEI/ARB/ARNI, β-blockers, MRA, vasodilators, inotropes) at baseline was higher in patients with eGFR between 60.0–89.9 and 30.0–59.9.

### Levosimendan infusion

In this study, levosimendan administration varied according to centers. In total of 56 patients (7%) received loading doses (ranging from 2.5–20 μg/kg) and intravenously pumped or dropped within 5–15 min. The maintenance dose was 0.013–0.6 μg·kg^−1^.min^−1^, and titrated by clinicians, based on symptoms, blood pressure, as well as adverse effects. The infusion time ranged from 300 to 3,750 min, with an average duration of 1,450 ± 307 min. Among those patients, 290 (36.8%) took levosimendan for more than 1,440 min, and levosimendan infusion was terminated earlier in 10 patients (1.1%) due to adverse events. The dose of levosimendan ranged from 1.5 mg to 25 mg, and mean dose was 11.84 ± 2.11 mg.

### Efficacy of levosimendan according to baseline eGFR level

**Figure 2:**
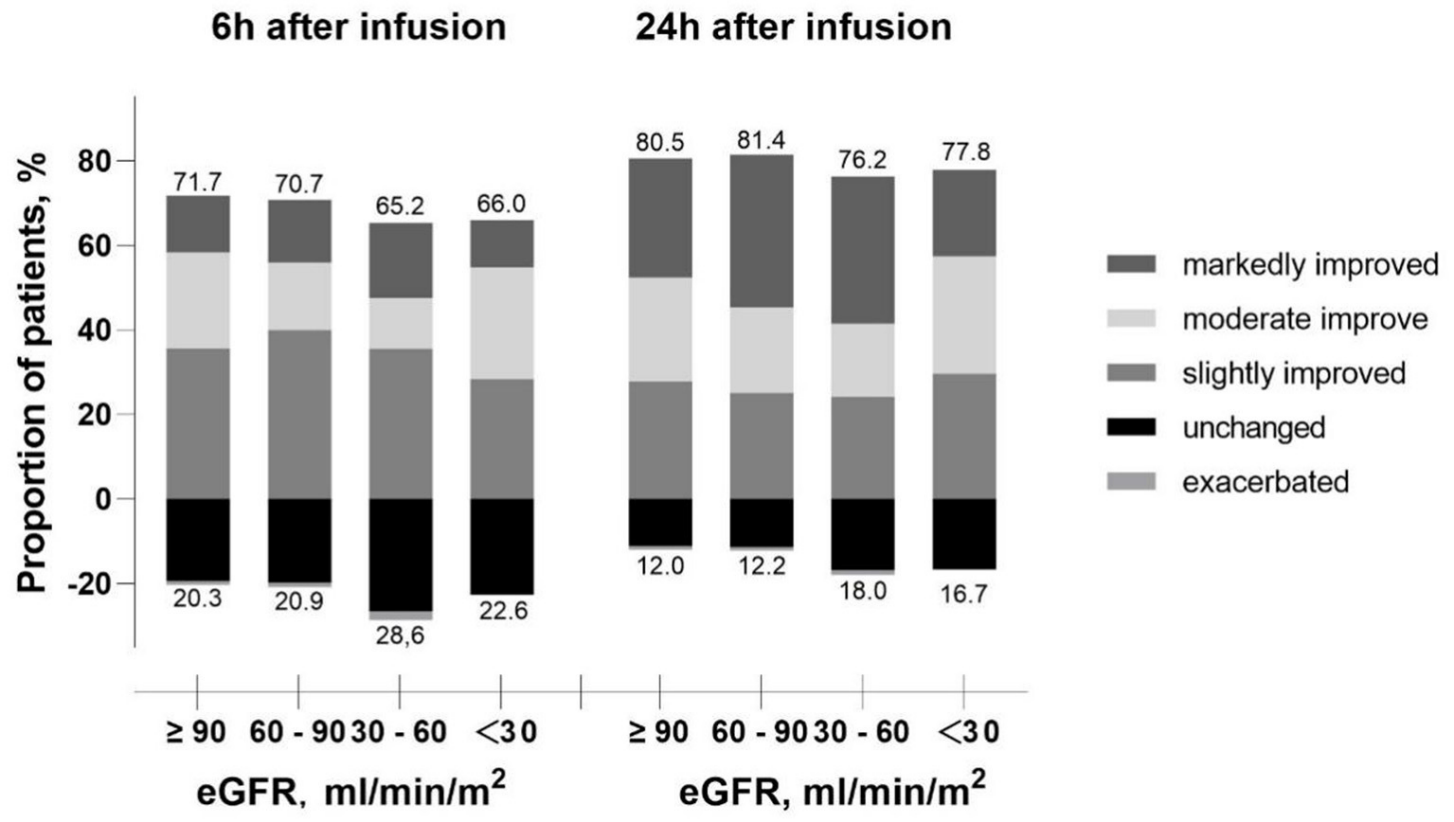
Proportion of patients improved or exacerbated over infusion time according to the eGFR level. eGFR, estimated glomerular filtration rate.

Among the 789 patients, the mean score of dyspnea was 3.0 ± 1.0 using 5-point Likert scale before levosimendan infusion. After 6 h infusion of levosimendan, dyspneaimprovedin 542 (68.7%) patients. And after treatment for 24 h, the efficacy rate increased up to 79.5% (627 patients). The efficacy rate from acute phase in patients treated with levosimendan was assessed in groups stratified by baseline eGFR level. Lower eGFR was associated with lower efficacy rate after 6 h infusion (71.7, 70.7, 65.2, and 66.0%, respectively) and after 24h infusion (80.5, 81.4, 76.2, and 77.8%, respectively) (Figure 2).

**Figure 3:**
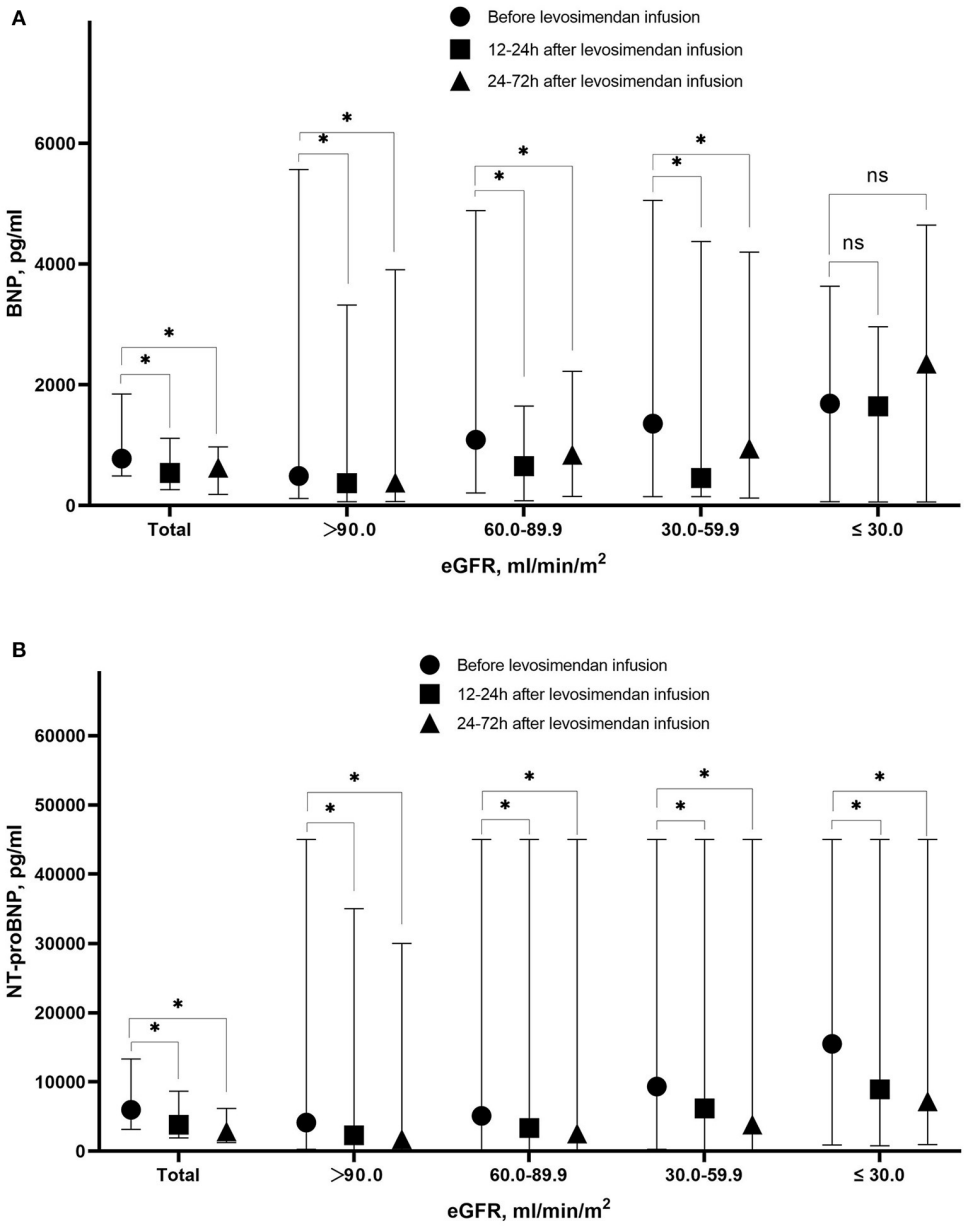
Level of BNP **(A)** and NT-pro BNP **(B)** before levosimendan infusion, at 12–24 h after levosimendan infusion, and 24–72 h after levosimendan infusion according to eGFR level. BNP, Brain natriuretic peptide; NT-pro BNP, N-terminal pro- brain natriuretic peptide; eGFR, estimated glomerular filtration rate. ^*^Statistically significant difference compared with eGFR level before levosimendan infusion (*p* < 0.05). ns Non-statistically significant difference compared with eGFR level before levosimendan infusion (*p* > 0.05).

With decreasing eGFR, significant increase of BNP or NT-pro BNP level was observed before levosimendan treatment, at 12–24 h and at 24–72 h after levosimendan infusion. Till 24–72 h after levosimendan infusion, significant decreases of NT-pro BNP levels from baseline in response to levosimendan treatment in each eGFR group. However, the trend of BNP levels was not significant in different eGFR groups (Figure 3).

### Changes of eGFR after levosimendan treatment according to baseline eGFR level

**Figure 4:**
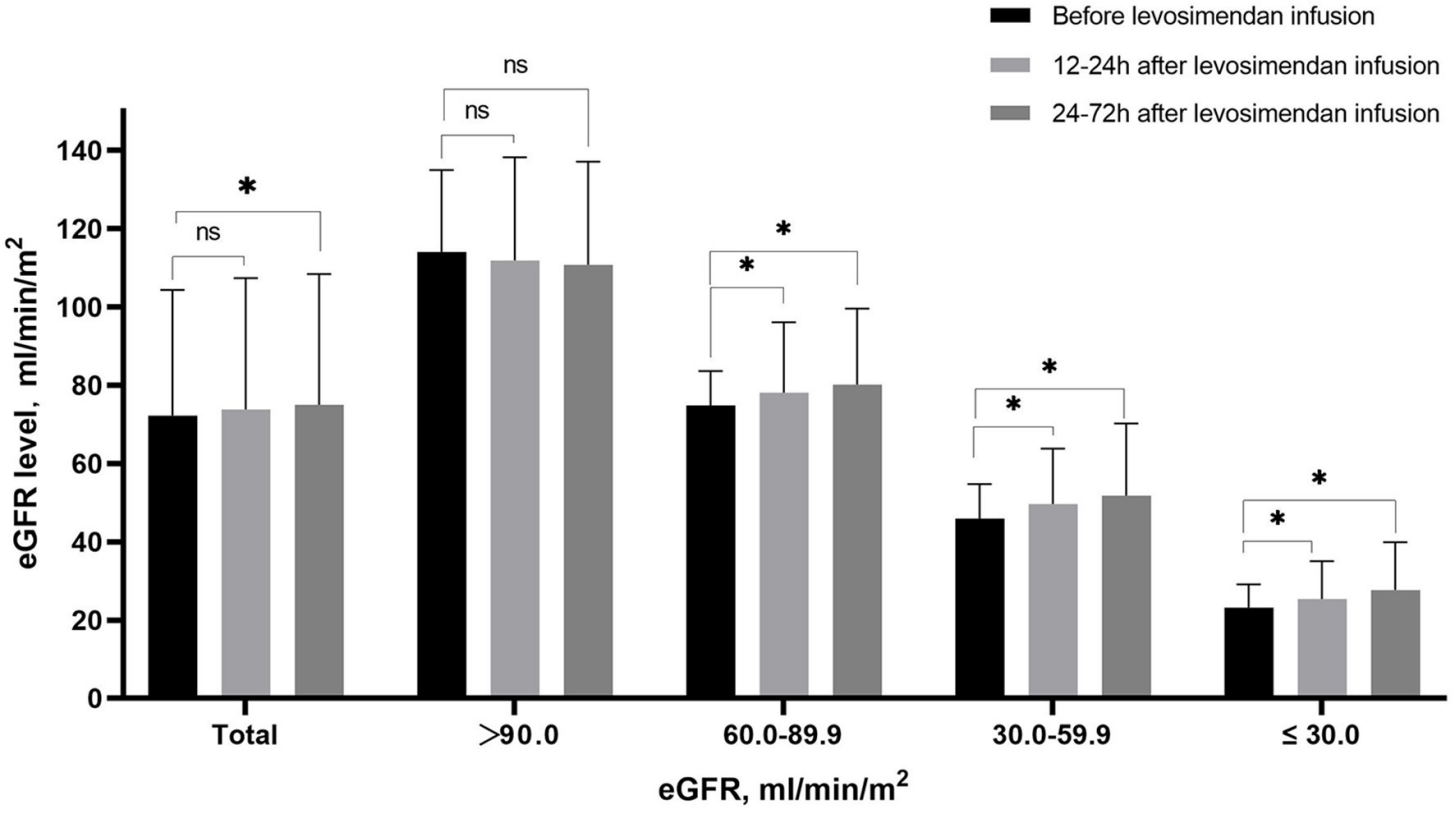
Mean eGFR before levosimendan infusion, at 12–24 h and 24–72 h after levosimendan infusion according to baseline eGFR level. ^*^Statistically significant difference compared with eGFR level before levosimendan infusion (*p* < 0.05). ns Non-statistically significant difference compared with eGFR level before levosimendan infusion (*p* > 0.05).

Levels of eGFR increased from baseline (72.6 ± 32.5 ml/min/m^2^) to 12–24 h (73.8 ± 33.5 ml/min/m^2^) and 24–72 h (75.0 ± 33.4 ml/min/m^2^) after starting treatment (*p* < 0.001). As shown in Figure 4, the eGFR levels increased after levosimendan infusion in patients with eGFR lower than 90.0 ml/min/m^2^. On the contrary, levosimendan infusion did not result in an increase in eGFR level in patients with the highest baseline eGFR levels.

### Safety

Adverse events occurred in 74 patients (9.3%), which was no significant differences among the eGFR groups (*p* = 0.85). Among those, 47 patients suffered hypotension (5.9%), and new onset arrhythmias, including atrial fibrillation or ventricular tachycardia, occurred in 15 patients (1.9%). Other observed potential adverse events included headache, nausea, vomiting and pruritus occurred in 9 patients (1.1%). In those with eGFR lower than 30 ml/min/m2, 5 patients suffered from adverse events, including hypotension (1 case) and new onset arrhythmia (4 cases), respectively.

## Discussion

In the present study, we firstly investigate the short- term efficacy and renoprotective effect of levosimendan in real-world AHF patients according to different renal function. Epidemiological investigations revealed that renal dysfunction was common in AHF patients who received levosimendan, 39.6% patients had eGFR lower than 60 mL/min/1.73 m^2^. The main finding was that levosimendan treatment improved the degree of dyspnea at 6 h and 24 h after infusion, especially in AHF patients with higher eGFR level. Moreover, levosimendan treatment was associated with a significant decrease of BNP or NT-pro BNP levels, as well as an increase of eGFR levels. However, in patients with normal renal function (eGFR higher than 90 mL/min/1.73 m^2^), the increase of eGFR was non-significant.

Levosimendan is a new positive inotropic drug with vasodilating properties by opening the KATP channels that have been extensively investigated and recommended to use in various clinical conditions (1, 12). In this registry, we describe the clinical characteristics and treatment of AHF patients who were treated with levosimendan, all these data reflect real-world information of these population. Consistent with other studies (13, 14), the baseline characteristics and treatment during hospital stay were similar. So far, levosimendan has been proved to reduce clinical signs and symptoms, and improve hemodynamics in RCTs (15, 16). And in present study, AHF patients also showed significant improvement in degree of dyspnea, and BNP or NT-pro BNP levels after levosimendan infusion, regardless of etiologies. However, in patients with higher eGFR, the proportions of efficacy rate were lower. It is speculated that AHF patients with higher eGFR had more comorbidities, more severe of AHF defined by NYHA or Killip classification and degree of dyspnea, which influences the treatment and outcomes (2). Furthermore, we found that levosimendan infusion could reduce BNP or NT-pro BNP levels in overall population as previous studies (17). In subgroup analysis, the difference of BNP levels between pre- and post- levosimendan infusion was non-significant among patients with eGFR lower than 30 ml/min/m^2^, it's might because few patients had examined BNP levels in this eGFR category.

On the other hand, previous experiments have reported that the use of levosimendan can improve cardiac output, urine amount and eGFR (6, 8, 9, 18, 19), many are small and characterized by heterogeneities (20). In accordance with these previous data (10), our study showed that levosimendan infusion caused a rapid and obvious improvement in eGFR levels at 24–72 h after starting treatment, compared with baseline values. However, the statistically significant effect sizes are small (75.0 vs. 72.6 ml/min/m^2^) and it remains to be seen whether such an increase in eGFR can be clinically relevant.

To the best of our knowledge, we firstly investigate the renoprotective effect of levosimendan according to different renal function. Interestingly, the renoprotective effect was non-significant in AHF patients with normal renal function (eGFR higher than 90 ml/min/m^2^). In our opinion, there may be several potential reasons for this phenomenon. Firstly, the multiple mechanisms contribute to kidney damage in AHF patients, including hypoperfusion due to hypotension and low cardiac output, renal venous congestion, interstitial fibrosis, tubular damage, and nephron loss linked to neurohormonal activation and inflammation (6). Levosimendan induced not only elevation of cardiac output, but also a selective dilation of preglomerular afferent arterioles to increase renal blood flow, even before its inotropic action (9). Therefore, in AHF patients with renal dysfunction, the mechanisms and renoprotective were more evident. Secondly, levosimendan has a short half-life (about 1.5 h), and the prolonged actions (about 7–9 days) are mainly due to its active metabolite OR-1896 (21). In patients with severe chronic renal failure, the half-life of OR-1896 was prolonged 1.5-fold (96.5 ± 19.5 h) in patients as compared with healthy subjects (61.6 ± 5.2 h) (22). So that, depressed eGFR reduced the renal excretion of levosimendan and increased concentration of levosimendan and OR-1896, which may lead to stronger inotropic and renoprotective effects.

Indeed, to avoid possible side effects, such as fatal arrhythmia, previous RCTs about levosimendan had excluded patients with an eGFR lower than 30 ml/min/m^2^, and use of levosimendan is not recommended by opinion consensus (5, 12), as well as by administration. However, in this real-world registry, AHF patients frequently accompanied with different severities renal dysfunction. And levosimendan has been used even in those with eGFR lower than 30 ml/min/m^2^ (6.8%), with similar incidence of adverse events, compared with other eGFR groups. Our findings advocate that severe renal dysfunction may amelioration after levosimendan infusion and not be a contraindication for using this inodilator. However, further investigation is needed to verify its efficacy and safety in AHF patients with eGFR lower than 30 ml/min/m^2^.

Despite a large population included in this observational registry, several limitations should be considered. First, this study did not have a control group, and patients with previous renal disease or DM were included. These methodological problems could result in confounding factors and affect the conclusion, given baseline characteristics, such as age, gender, comorbidities, etc., and other treatment measurements, would also affect the renal function. Second, patients were not consecutively enrolled during the whole course of the study, which might lead to a selection bias. Third, the degree of dyspnea was self-report before and after levosimendan infusion, and the hemodynamics parameters were not always assessed. Fourth, we did not directly measure the GFR by specific tracers, such as inulin and iothalamate, which are not commonly carried out in the clinical practice, and eGFR may not be the most accurate and sensitive parameter for detecting early and rapid changes in renal function (23). Additionally, we did not measure other renal hemodynamic and urine parameters that may be useful to explain these results. Therefore, well-designed studies are needed to verify the conclusions and explore the underlying mechanism behind these findings.

## Conclusion

In AHF patients who received levosimendan, degree of dyspnea and levels of BNP or NT-pro BNP were significantly improved, especially in patients with high eGFR levels. Furthermore, levosimendan infusion increase eGFR only in AHF patients with renal dysfunction.

## Data availability statement

The raw data supporting the conclusions of this article will be made available by the authors, without undue reservation.

## Ethics statement

The studies involving human participants were reviewed and approved by Fuwai Hospital. The patients/participants provided their written informed consent to participate in this study.

## Author contributions

HZ collected the data, performed the statistical analysis, drafted, and wrote the manuscript. Y-mY designed the study and revised the manuscript. LJ, RF, PQ, XZ, TT, and G-xF collected the data. All authors read and approved the final manuscript.

## Conflict of interest

The authors declare that the research was conducted in the absence of any commercial or financial relationships that could be construed as a potential conflict of interest.

## Publisher's note

All claims expressed in this article are solely those of the authors and do not necessarily represent those of their affiliated organizations, or those of the publisher, the editors and the reviewers. Any product that may be evaluated in this article, or claim that may be made by its manufacturer, is not guaranteed or endorsed by the publisher.

## References

[1] McDonagh TA Metra M Adamo M Gardner RS Baumbach A Bohm M. 2021 ESC Guidelines for the diagnosis and treatment of acute and chronic heart failure. Eur Heart J. (2021) 42:3599–726. 10.1093/eurheartj/ehab368 34447992

[2] Heywood JT Fonarow GC Costanzo MR Mathur VS Wigneswaran JR Wynne J. High prevalence of renal dysfunction and its impact on outcome in 118,465 patients hospitalized with acute decompensated heart failure: a report from the ADHERE database. J Card Fail. (2007) 13:422–30. 10.1016/j.cardfail.2007.03.011 17675055

[3] Rangaswami J Bhalla V Blair JE Chang TI Costa S Lentine KL. Cardiorenal syndrome: classification, pathophysiology, diagnosis, and treatment strategies: a scientific statement from the American Heart Association. Circulation. (2019) 139:e840–e78. 10.1161/CIR.0000000000000664 30852913

[4] DesJardin JT Teerlink JR. Inotropic therapies in heart failure and cardiogenic shock: an educational review. Eur Heart J Acute Cardiov Care. (2021) 10:676–86. 10.1093/ehjacc/zuab047 34219157

[5] Bouchez S Fedele F Giannakoulas G Gustafsson F Harjola VP Karason K. Levosimendan in acute and advanced heart failure: an expert perspective on posology and therapeutic application. Cardiov Drugs Ther. (2018) 32:617–24. 10.1007/s10557-018-6838-2 30402660 PMC6267661

[6] Follath F Cleland JG Just H Papp JG Scholz H Peuhkurinen K. Efficacy and safety of intravenous levosimendan compared with dobutamine in severe low-output heart failure (the LIDO study): a randomised double-blind trial. Lancet. (2002) 360:196–202. 10.1016/S0140-6736(02)09455-2 12133653

[7] Guerrero-Orriach JL Malo-Manso A Ramirez-Aliaga M Florez Vela AI Galán-Ortega M Moreno-Cortes I. Renal and neurologic benefit of levosimendan vs dobutamine in patients with low cardiac output syndrome after cardiac surgery: clinical Trial FIM-BGC-2014-01. Front Pharmacol. (2020) 11:1331. 10.3389/fphar.2020.01331 32982742 PMC7479222

[8] Lannemyr L Ricksten SE Rundqvist B Andersson B Bartfay SE Ljungman C. Differential effects of levosimendan and dobutamine on glomerular filtration rate in patients with heart failure and renal impairment: a randomized double-blind controlled trial. J Am Heart Assoc. (2018) 7:e008455. 10.1161/JAHA.117.008455 30369310 PMC6201411

[9] Fedele F Bruno N Brasolin B Caira C D'Ambrosi A Mancone M. Levosimendan improves renal function in acute decompensated heart failure: possible underlying mechanisms. Eur J Heart Fail. (2014) 16:281–8. 10.1002/ejhf.9 24464960

[10] Hou ZQ Sun ZX Su CY Tan H Zhong X Hu B. Effect of levosimendan on estimated glomerular filtration rate in hospitalized patients with decompensated heart failure and renal dysfunction. Cardiovasc Ther. (2013) 31:108–14. 10.1111/1755-5922.12001 23490237

[11] Ma YC Zuo L Chen JH Luo Q Yu XQ Li Y. Modified glomerular filtration rate estimating equation for Chinese patients with chronic kidney disease. Journal of the American Society of Nephrology: JASN. (2006) 17:2937–44. 10.1681/ASN.2006040368 16988059

[12] Tycińska A Gierlotka M Bugajski J Deja M Depukat R Gruchała M. Levosimendan in the treatment of patients with acute cardiac conditions: an expert opinion of the Association of Intensive Cardiac Care of the Polish Cardiac Society. Kardiol Pol. (2020) 78:825–34. 10.33963/KP.15551 32788567

[13] Zhang Y Zhang J Butler J Yang X Xie P Guo D. Contemporary epidemiology, management, and outcomes of patients hospitalized for heart failure in China: Results from the china heart failure (China-HF) registry. J Card Fail. (2017) 23:868–75. 10.1016/j.cardfail.2017.09.014 29029965

[14] Wang GG Wang SJ Qin J Li CS Yu XZ Shen H. Characteristics, Management, and Outcomes of Acute Heart Failure in the Emergency Department: A Multicenter Registry Study with 1-year Follow-up in a Chinese Cohort in Beijing. Chin Med J. (2017) 130:1894–901. 10.4103/0366-6999.211880 28776539 PMC5555121

[15] Pashkovetsky E Gupta CA Aronow WS. Use of levosimendan in acute and advanced heart failure: short review on available real-world data. Ther Clin Risk Manag. (2019) 15:765–72. 10.2147/TCRM.S188761 31354283 PMC6588712

[16] Packer M Colucci W Fisher L Massie BM Teerlink JR Young J. Effect of levosimendan on the short-term clinical course of patients with acutely decompensated heart failure. JACC Heart failure. (2013) 1:103–11. 10.1016/j.jchf.2012.12.004 24621834

[17] Cui D Liao Y Li G Chen Y. Levosimendan Can Improve the Level of B-Type Natriuretic Peptide and the Left Ventricular Ejection Fraction of Patients with Advanced Heart Failure: A Meta-analysis of Randomized Controlled Trials. Am J Cardiov Drugs. (2021) 21:73–81. 10.1007/s40256-020-00416-y 32462455

[18] Grossini E Molinari C Pollesello P Bellomo G Valente G Mary D. Levosimendan protection against kidney ischemia/reperfusion injuries in anesthetized pigs. J Pharmacol Exp Ther. (2012) 342:376–88. 10.1124/jpet.112.193961 22566668

[19] Bragadottir G Redfors B Ricksten SE. Effects of levosimendan on glomerular filtration rate, renal blood flow, and renal oxygenation after cardiac surgery with cardiopulmonary bypass: a randomized placebo-controlled study. Crit Care Med. (2013) 41:2328–35. 10.1097/CCM.0b013e31828e946a 23921271

[20] Yilmaz MB Grossini E Silva Cardoso JC Édes I Fedele F Pollesello P. Renal effects of levosimendan: a consensus report. Cardiov Drugs Ther. (2013) 27:581–90. 10.1007/s10557-013-6485-6 23929366 PMC3830192

[21] Banfor PN Preusser LC Campbell TJ Marsh KC Polakowski JS Reinhart GA. Comparative effects of levosimendan, OR-1896, OR-1855, dobutamine, and milrinone on vascular resistance, indexes of cardiac function, and O2 consumption in dogs. Am J Physiol Heart Circ Physiol. (2008) 294:H238–48. 10.1152/ajpheart.01181.2007 17982006

[22] Puttonen J Kantele S Kivikko M Häkkinen S Harjola VP Koskinen P. Effect of severe renal failure and haemodialysis on the pharmacokinetics of levosimendan and its metabolites. Clin Pharmacokinet. (2007) 46:235–46. 10.2165/00003088-200746030-00004 17328582

[23] Ahmad T Jackson K Rao VS Tang WHW Brisco-Bacik MA Chen HH. Worsening renal function in patients with acute heart failure undergoing aggressive diuresis is not associated with tubular injury. Circulation. (2018) 137:2016–28. 10.1161/CIRCULATIONAHA.117.030112 29352071 PMC6066176

